# A Small Randomized Controlled Pilot Trial Comparing Mobile and Traditional Pain Coping Skills Training Protocols for Cancer Patients with Pain

**DOI:** 10.1155/2016/2473629

**Published:** 2016-11-06

**Authors:** Tamara J. Somers, Sarah A. Kelleher, Kelly W. Westbrook, Gretchen G. Kimmick, Rebecca A. Shelby, Amy P. Abernethy, Francis J. Keefe

**Affiliations:** ^1^Department of Psychiatry and Behavioral Sciences, Duke University Medical Center, Durham, NC, USA; ^2^Department of Internal Medicine, Duke Cancer Institute, Duke University Medical Center, Durham, NC, USA; ^3^Duke University School of Medicine, Durham, NC, USA

## Abstract

Psychosocial pain management interventions are efficacious for cancer pain but are underutilized. Recent advances in mobile health (mHealth) technologies provide new opportunities to decrease barriers to access psychosocial pain management interventions. The objective of this study was to gain information about the accessibility and efficacy of mobile pain coping skills training (mPCST) intervention delivered to cancer patients with pain compared to traditional in-person pain coping skills training intervention. This study randomly assigned participants (*N* = 30) to receive either mobile health pain coping skills training intervention delivered via Skype or traditional pain coping skills training delivered face-to-face (PCST-trad). This pilot trial suggests that mPCST is feasible, presents low burden to patients, may lead to high patient engagement, and appears to be acceptable to patients. Cancer patients with pain in the mPCST group reported decreases in pain severity and physical symptoms as well as increases in self-efficacy for pain management that were comparable to changes in the PCST-trad group (*p*'s < 0.05). These findings suggest that mPCST, which is a highly accessible intervention, may provide benefits similar to an in-person intervention and shows promise for being feasible, acceptable, and engaging to cancer patients with pain.

## 1. Introduction

In patients with cancer, persistent pain is related to poor physical functioning, increased physical symptoms, and low overall health-related quality of life [1, 2]. High pain level in cancer patients also is associated with decreased survival time and can be a significant predictor of survival time [3, 4]. Patients with cancer report pain to be their most distressing symptom [5, 6]. While psychosocial pain management interventions have shown efficacy for cancer patients, patient access to such interventions is a significant challenge. Most psychosocial pain management interventions are delivered through in-person sessions at large tertiary medical centers. For patients with cancer and pain, there are several barriers to in-person sessions [7].

Mobile health (mHealth) technology advances may decrease a number of the barriers to psychosocial cancer pain interventions. First, patients with chronic diseases report being too busy or not having enough time to attend in-person sessions; mHealth technology can make intervention delivery available at a time and place convenient for the patient [8]. Second, patients with chronic disease report not feeling well, emotional distress, and problems with transportation (e.g., cost) as barriers to accessing psychosocial interventions [8, 9]; mHealth technology decreases the physical burdens associated with in-person appointments and can eliminate cost of travel. Third, patients report that attributes of an acceptable intervention include convenience, appropriateness, and effectiveness of the intervention [10–12]; mHealth technologies can be used to increase the availability of interventions that are convenient, acceptable to patients, and effective.

We recently conducted a single-arm pilot trial that examined the feasibility, acceptability, and value of mHealth psychosocial pain management intervention—mobile pain coping skills training (mPCST) [13]. In this trial (*N* = 25), breast, lung, prostate, and colorectal cancer patients with pain participated in a four-session mPCST protocol using video conferencing on a tablet computer while in their home environment. Feasibility was demonstrated by a high average number of intervention sessions completed (3.36 sessions out of a possible 4) and 84% overall trial completion. Participants reported mPCST to be a highly acceptable intervention using a standardized satisfaction questionnaire and 95% reported that mPCST improved their pain management. Participants receiving the mPCST intervention also reported lower levels of pain severity, physical symptoms, psychological distress, and pain catastrophizing from the period before to the period after intervention.

Building on this single-arm pilot trial, the primary objective of the current small pilot randomized controlled trial was to gain additional information about the accessibility and efficacy of mPCST delivered to cancer patients when compared to traditional in-person pain coping skills training intervention. We hypothesized the following: (1) compared to PCST-trad, mPCST would improve intervention access as assessed by feasibility (i.e., attrition, adherence, and completion time), patient's self-report of burden related to intervention, patient intervention engagement (i.e., use of skills), and self-report of a standardized acceptability scale and (2) mPCST would lead to significant decreases in pain, pain catastrophizing, physical functioning, physical symptoms, and psychological distress and despair and increases in self-efficacy for pain management comparable to PCST-trad.

## 2. Materials and Methods

### 2.1. Study Design

**Figure 1:**
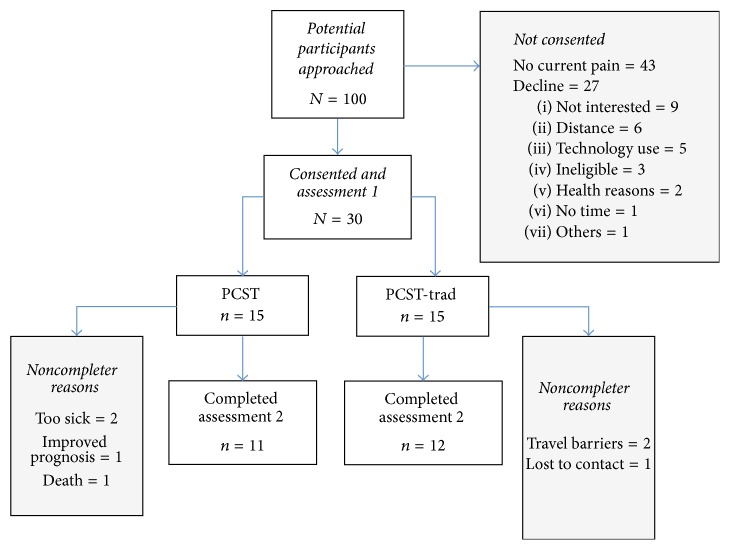


This small randomized controlled pilot trial randomly assigned participants (*N* = 30) to either mPCST or PCST-trad. Pre- (prior to randomization) and postintervention assessments included measures of pain, pain catastrophizing, self-efficacy for pain management, physical functioning, physical symptoms, and psychological distress and despair. Patient burden, acceptability, and engagement were also assessed after intervention. The preintervention assessment was completed with the study team at the participant's first visit and the postintervention assessment was completed one to two weeks following their final study intervention session. Participants completed both pre- and postintervention assessments using a tablet computer and were compensated $20 per assessment for a total of $40. All participants continued to receive their usual medical care. Institutional review board approved the study.  Figure 1 provides a chart of recruitment, consent, and completion.

### 2.2. Participants

Participants in this study were diagnosed with breast, prostate, lung, or colorectal cancer. All participants had a clinical pain score of 3 or greater [14] (on a scale of 0 = “no pain” to 10 = “pain as bad as you can imagine”) recorded in their medical record on two occasions > three weeks apart but < 12 months apart. To be included, patients had to be at least 18 years old and have a life expectancy of at least 6 months as assessed by their oncologist. Exclusion criteria included metastases to the brain, treatment for a serious psychological disorder (e.g., schizophrenia) in the last 6 months, and prior engagement in cancer pain coping skills training protocol.

### 2.3. Intervention Conditions

#### 2.3.1. Mobile Health Pain Coping Skills Training

Participants randomized to this condition were provided with an iPad to take home with them to participate in mPCST video-conferencing sessions using Skype. Skype sessions were delivered by a Ph.D.-level clinical psychologist or an advanced clinical psychology student/intern closely supervised by a licensed Ph.D.-level clinical psychologist. Sessions for both the mPCST and PCST-trad conditions were conducted in an individual format and were scheduled based on patient and provider availability; however, the therapist always worked to schedule at times most convenient to the patient. mPCST Skype sessions were delivered by the therapist in the medical center to the patient in his or her own home likely increasing flexibility and ease of scheduling as mPCST patients did not have to leave their home.

Session content was based on pain coping skills training (PCST) intervention developed by Keefe et al. [15]. PCST uses cognitive-behavioral theory principles to teach patients coping skills that can enhance their pain management. The 4-session mPCST in this study was carefully designed to include pain coping skills that have shown efficacy in patients with cancer pain. In each session, participants were taught a skill intended to increase their ability to manage their pain and then given the assignment of practicing with that skill prior to the next session. Session 1 included pain education focused on the gate control theory of pain [16] to promote understanding that pain is a complex experience influenced by thoughts, feelings, and behaviors; progressive muscle relaxation (PMR) was taught as a strategy to decrease bodily tension and subjective distress. Session 2 included two skills. Activity-rest cycling was provided as a strategy to pace activities so as to gradually increase involvement in daily activities that were painful. Pleasant activity scheduling involved helping participants identify activities they enjoy that they have stopped doing due to pain (e.g., seeing friends) and set goals for new activities they may enjoy trying (e.g., walking, going out to dinner, and visiting friends). Session 3 taught the skill of cognitive restructuring which involves examining overly negative thoughts about pain (e.g., I can not do anything I used to do) that lead to negative consequences (e.g., feeling depressed, angry) and shifting negative thoughts to be more neutral or positive (e.g., I cannot do everything I used to, but I can still do some things I enjoy). Session 4 taught participants the skill of imagery for relaxation and distraction and brief applied relaxation exercise (the minirelaxation practice).

#### 2.3.2. Traditional Pain Coping Skills Training 

Participants in this condition received the same 4 sessions and content as participants in the mPCST condition, but all of the training was conducted at in-person sessions at the medical center.

### 2.4. Measures

#### 2.4.1. Feasibility

We assessed feasibility by examining study attrition (i.e., study completion), adherence (i.e., completed intervention sessions and assessments), and completion time for each study condition (i.e., days from session 1 to session 4).

#### 2.4.2. Patient Burden

Patient burden was measured by assessing 13 burden areas associated with accessing care following a cancer diagnosis. Items were selected based on information gathered from focus groups asking cancer survivors about barriers and burdens to accessing cancer care. Patients rated on a scale from 1 = “not at all” to 4 = “very much” how difficult it was to complete the study intervention sessions based on each burden. Scale 1 (Symptom Burdens) included items (factor loadings) asking about fatigue (0.73), pain (0.68), time (0.64), anxiety (0.67), feeling down or blue (0.53), and feeling stressed and overwhelmed (0.57). Scale 2 (Practical Burdens) included items on distance (0.51) and difficulties getting to session (0.98). Items asking about the cost of sessions, finding transportation to sessions, taking time off work, finding childcare, and forgetting about sessions had small factor loadings and they were dropped from analyses. The correlation between the two factor scales was *r* = 0.10 and scale reliability was 0.78 and 0.59, respectively.

#### 2.4.3. Patient Engagement

Patient engagement was assessed at the postintervention assessment. Participants were asked how many days in the last seven days they had used each of the pain coping skills. They were also asked how often since their final intervention session they had used the pain coping skills or ideas taught on this scale 0 = “not at all” to 4 = “almost every day.”

#### 2.4.4. Patient Acceptability

Acceptability was assessed at the postintervention assessment with the Client Satisfaction Questionnaire 10-item version [17]. Sample questions include the following: “Did you get the kind of information you wanted?”; “Would you recommend this program to a friend with pain?”; and “Did this program help you to deal more effectively with the pain you experience due to cancer?”; this questionnaire contained 10 items rated from 1 = “low acceptability” to 4 = “high acceptability.” Summing patient responses created scores. We also examined the individual item asking participants about the quality of the program rated 1 = “poor” to 4 = “excellent.”

#### 2.4.5. Pain Severity

Pain severity was assessed at the period before and the period after intervention with the Brief Pain Inventory (BPI) [18]. The BPI asked patients to assess their worst, least, average, and current pain, where 0 = “no pain” and 10 = “pain as bad as you can imagine.” Applicable items were reference to the last 7 days. Items were averaged for the pain severity score. This measure has been recommended for use in all chronic pain clinical trials [19, 20]. Internal consistency was good in this sample (Cronbach's alpha = 0.82–0.84).

#### 2.4.6. Pain Catastrophizing

Pain catastrophizing was assessed at the period before and the period after intervention with the 6-item pain catastrophizing scale of the Coping Strategies Questionnaire [21]. These items ask about participants' tendency to catastrophize when faced with pain and are answered on a scale of 0 = “never” to 6 = “always.” Items are summed. This scale has good reliability in cancer patients [22] and had good reliability in this sample (Cronbach's alpha = 0.90–0.93).

#### 2.4.7. Pain Self-Efficacy

Pain self-efficacy was assessed at the period before and the period after intervention with the pain self-efficacy subscale of the Chronic Pain Self-Efficacy Scale [23]. This subscale contains 5 items that inquire about participants: certainty about degree of pain control, pain during daily activities, controlling pain during sleep, and making pain reductions without extra medication. Items were answered on a scale of 10 = “very uncertain” to 100 = “very certain.” An average of the items was used as the final score. This scale has shown good reliability in cancer patients [19, 23] and evidenced fair reliability in this sample (Cronbach's alpha = 0.71–0.80).

#### 2.4.8. Physical Functioning

Physical functioning was assessed at the period before and the period after intervention with the 4-item physical functioning scale of the Patient Care Monitor (PCM) v2 [20, 24]. The PCM has been validated against other standard symptom inventories and quality-of-life scales. Physical functioning items asked about participants' ability to run, do light physical work or fun activities, do hard physical work or fun activities, and function normally, referencing the last 7 days. Patients rate their responses: 0 = “not a problem” to 10 = “as bad as possible.” Higher scores represent lower physical functioning; internal consistency was good in this sample (Cronbach's alpha = 0.83–0.84).

#### 2.4.9. Physical Symptoms

Physical symptoms were assessed at the period before and the period after intervention with the physical symptom scale of the PCM [20, 24]. The physical symptoms subscale has five items, which ask about patients' fatigue, concentration, pain, sleepiness, and insomnia. Responses were on a scale of 0 = “not a problem” to 10 = “as bad as possible.” Internal consistency in this sample was fair to good (Cronbach's alpha = 0.73–0.86).

#### 2.4.10. Psychological Distress

Psychological distress was assessed at the period before and the period after intervention with 4 items from the PCM asking about crying or feeling like crying, being worried, feeling nervous, tense, or anxious, and feeling sad or depressed [20, 24]. Items referenced the last 7 days and were scored on a scale of 0 = “not a problem” to 10 = “as bad as possible.” Internal consistency was good (Cronbach's alpha = 0.83–0.89).

#### 2.4.11. Psychological Despair

Despair was assessed at the period before and the period after intervention with 6 items from the PCM asking about hopelessness, lost interest in people and activities, helplessness, guilt, and worthlessness [20, 24]. Items referenced the last 7 days and were scored on a scale of 0 = “not a problem” to 10 = “as bad as possible.” Internal consistency was good in this sample (Cronbach's alpha = 0.85–0.91)

#### 2.4.12. Demographic and Medical Variables

Participants' age, gender, race, cancer type, diagnosis date, and cancer stage at study entry were collected through the patients electronic medical record. Participants' marital status, education level, comorbid medical disorders, and cancer treatments in the last week were collected through patient self-report at the preintervention assessment.

### 2.5. Statistical Analyses

Descriptive analyses were computed for patient demographic (i.e., age, gender, race, education, and marital status) and medical variables (i.e., cancer type, months since diagnosis, cancer stage at study entry, comorbid disorders, and cancer treatments in the last week) (Table 1). We examined the rates and/or descriptive data related to attrition, adherence, completion time, patient burden, and patient engagement. *t*-tests were used to examine if there were group differences in days to completion, patient burden, and patient engagement. Pearson or point biserial correlations were used to examine the relationships between demographic/medical information and study variables of interest. We used repeated measures analysis of variance (ANOVA) to examine intervention related changes.

## 3. Results

30 participants with breast, lung, prostate, and colorectal cancer were enrolled in this trial; 15 were randomized to each condition. Participants were M = 60 (SD = 11) years old, 50% were female, 80% reported being partnered, and most participants were White (97%). Descriptive information is summarized in Table 1.

Table 2 examines the preintervention bivariate relationships between demographic/medical variables and the study variables of interest (i.e., pain intensity, pain catastrophizing, pain self-efficacy, physical functioning, physical symptoms, distress, and despair). Pain intensity was associated with age (*r* = −0.43; *p* < 0.05), suggesting that younger participants reported higher levels of pain. Psychological distress was associated with gender (*r* = 0.49; *p* < 0.01), suggesting women reported more psychological distress compared to men. Psychological despair was associated with cancer stage (*r* = 0.38; *p* < 0.05) and number of comorbid medical disorders (*r* = 0.40; *p* < 0.05). These relationships suggest that higher levels of psychological despair are associated with more advanced cancer and more comorbid disorders. Race was not examined in these analyses as the majority of participants were White (97%).

### 3.1. Feasibility

#### 3.1.1. Attrition

Thirty participants were enrolled into the study and randomized. Of these 30 participants, 4 (3 mPCST, 1 PCST-trad) participants never started the study intervention. An additional 3 (1 mPCST, 2 PCST-trad) participants did not complete the second study assessment. Reasons for the 7 noncompleters included the following: cancer progression and/or less than 1-month life expectancy (2 mPCST), death (1 mPCST), improved prognosis (1 mPCST), not traveling to cancer center (1 PCST-trad), traveling too far (1 PCST-trad), and being lost to contact (1 PCST-trad). It is possible that this small trial (*N* = 30) may not have had enough participants to detect equal events such as disease progression or death across groups; participants in the mPCST group were more likely to report non-study completion due to disease progression and in one case, death. Of note, disease status at study entry by group was as follows: Stage 0 (mPCST = 0, mPCST-trad = 1), Stage 1 (mPCST = 2, mPCST-trad = 2), Stage 2 (mPCST = 2, mPCST-trad = 3), Stage 3 (mPCST = 5, mPCST-trad = 3), and Stage 4 (mPCST = 6, mPCST-trad = 6). The reasons for noncompleters in the PCST-trad group may have been due to the constraints of an in-person intervention (e.g., that it is too far to travel).

#### 3.1.2. Adherence

Participants in both conditions who completed the study (*n* = 23) participated in an average of 3.83 (SD = 0.65) out of 4 possible sessions. mPCST study completers (*n* = 11) participated in an average of 3.64 (SD = 0.92) study intervention sessions, with 9 participants completing all sessions. One mPCST participant completed only one study session but agreed to complete the follow-up assessment; another mPCST participant completed 3 out of the 4 sessions. The 12 PCST-trad study completers participated in all four study intervention sessions.

#### 3.1.3. Intervention Completion Time

Among all study participants (*n* = 21) that completed all four intervention sessions, the average time from session 1 to session 4 was 46.81 (SD = 28.46) days. mPCST patients (*n* = 9) took an average of 29.67 (SD = 11) days and PCST-trad participants (*n* = 11) took an average of 59.67 (SD = 31.01) days. The average number of days to completion was significantly different between groups (*t*(19) = 2.76; *p* = 0.01).

### 3.2. Patient Burden

Two subscales of burdens (Symptom, Practical) to intervention participation were created and examined. All participants scored an average of M = 1.51 (SD = 0.55) on the Symptom Burden scale and M = 1.37 (SD = 0.57) on the Practical Burden scale. The reported barriers to intervention were low and there were no differences between groups. The three barriers with the highest individual averages were being tired or fatigued (M = 2.04; SD = 1.15), pain (M = 2.04; 1.14), and distance from the medical center (M = 1.61; SD = 0.84).

### 3.3. Patient Engagement

At the postintervention assessment, all participants reported practicing the skills over the last 7 days as follows: progressive muscle relaxation with provided audio, M = 2.43 (SD = 1.95); progressive muscle relaxation without audio, M = 3.78 (SD = 2.54); activity-rest cycling, M = 4.17 (SD = 2.72); pleasant activity planning, M = 4.09 (SD = 2.13); cognitive restructuring, M = 4.77 (SD = 2.22); imagery, M = 4.13 (SD = 2.52); and minirelaxation, M = 3.70 (SD = 2.58). Following treatment, participants reported using pain coping skills learned in the intervention on average several days a week (M = 3.26, SD = 1.05; scale 0 = “not at all” to 4 = “almost every day”). There were no group differences reported in specific pain coping skills use or all skills use (*p*'s > 0.17).

### 3.4. Patient Acceptability

All participants rated the program to be highly acceptable with an average rating of 3.5 (SD = 0.59) (1 = “low acceptability” to 4 = “high acceptability”). There were no significant differences in acceptability between groups. Participants rated the pain coping skills program overall with 61% (*n* = 14) rating it excellent, 35% (*n* = 8) rating it good, and 4% (*n* = 1) rating it poor.

### 3.5. Pre- to Postintervention Changes

#### 3.5.1. Between-Group Pre- to Postintervention Effects

There were between-group differences for pain catastrophizing (*F*(1,20) = 14.23; *p* = 0.001) and physical disability (*F*(1,21) = 13.59; *p* = 0.001). Mean scores suggest that the PCST-trad group experienced decreases in pain catastrophizing and physical disability not seen in the mPCST group. The statistics reported are for the between-groups interaction. No other pre- to postintervention differences between groups were found.

#### 3.5.2. Main Pre- to Postintervention Effects

When examining the outcomes of interest across all participants, there were time effects for pain severity (*F*(1,19) = 6.43; *p* = 0.20), pain catastrophizing (*F*(1,20) = 25.93; *p* < 0.001), self-efficacy for pain management (*F*(1,21) = 6.44; *p* = 0.02), physical symptoms (*F*(1,20) = 4.72; *p* = 0.04), and physical disability (*F*(1,21) = 5.16; *p* = 0.03). Mean scores (Table 3) suggest all changes were in a beneficial direction. No time effects were seen for psychological distress or psychological despair.

## 4. Discussion

This study used a small, randomized controlled pilot trial to begin to examine the accessibility of mobile pain coping skills training intervention when compared to traditional in-person pain coping skills training intervention. Accessibility was examined in several ways including feasibility, patient burden, patient engagement, and acceptability. Evidence from this trial suggests that mPCST is an accessible intervention for cancer patients with pain; participants completed on average 3.83 out of 4 video-conferencing sessions. mPCST was also found to be feasible, present a low burden to patients, and have high patient engagement and to be acceptable to patients. We were also interested in examining the value of mPCST by examining pre- to postintervention changes in key pain-related outcomes and comparing these changes to those obtained in patients undergoing a traditional PCST program. Cancer patients with pain in the mPCST group reported decreases in pain severity and physical symptoms as well as increases in self-efficacy for pain management that were comparable to changes in the PCST-trad group, suggesting that mPCST may provide similar benefits to an in-person intervention.

Findings regarding the accessibility of mPCST provide important information about this intervention and suggest areas of future work. First, one of the most interesting findings was the statistically significant difference in completion time between mPCST and PCST-trad. Participants in the PCST-trad condition took twice as long to complete the four-session intervention (M = 60 days) as participants in the mPCST condition (M = 30 days). The pain coping skills training protocol in this study was designed and intended to be delivered weekly over four weeks. This finding may suggest that providing cancer patients with pain the option to receive pain coping skills training intervention in their home with mobile technology may increase treatment fidelity (e.g., delivery of intervention in the timeframe intended). It may be that, in a clinical setting where providers are faced with the challenges of timing, billing, and scheduling compared to this formal research trial in which we used multiple resources to retain patients, many of the PCST-trad participants would not have completed the 4-session in-person intervention. Second, we found that study attrition in the PCST-trad condition appeared to be due to difficulties with distance to the medical center as one participant reported they could not complete sessions because they were no longer traveling to the medical center, one reported it was too far to travel, and a third was lost to contact. Attrition in the mPCST randomization group was largely due to worsening disease prognosis/death that may not have been equally represented in both groups due to the small sample size. As shown in Figure 1, almost 10% of eligible patients who declined study participation cited that distance or possible travel to the medical center was the major factor for declining participation. Third, among participants who completed the study there were no differences between groups in adherence to study sessions, reported patient burden, or patient engagement in study skills practice. Interestingly, one of the top three reported patient burdens was distance to the medical center. No significant differences between groups were found in burdens, but it would be valuable to examine this variable in a larger trial.

We found that overall participants in this trial reported pre- to postintervention improvements in pain severity, pain catastrophizing, self-efficacy for pain management, physical symptoms, and physical disability. Participants in both groups experienced similar benefits in decreased pain and increased self-efficacy for pain management. This finding is significant for two reasons. First, patients with cancer often have progression in disease and/or receive intense cancer-related treatments that can lead to increased pain levels. The fact that patients in this study reported significant decreases in pain from the period before to the period after intervention may suggest that pain coping skills training protocols can help cancer patients with pain to improve their pain and pain coping even in the face of disease progression and cancer-related treatments. Second, self-efficacy for pain management has emerged as one of the most important psychosocial constructs in predicting patients' pain and their pain-related disability. Increasing patients' self-efficacy for pain management may be particularly important as cancer patients with pain often experience persistent pain even following cancer treatment (e.g., breast cancer patients on preventative hormone medication). Interestingly, our data suggests that PCST-trad led to greater decreases in pain catastrophizing and physical disability when compared to the mPCST group. This finding may suggest that the mPCST protocol did not target these outcomes as well as a traditional in-person intervention and that further iterations of the intervention protocol should be altered to more intensely intervene in these areas.

This study is a small, randomized pilot trial and future work with larger sample sizes is necessary. This work has several limitations. This study is limited by the small sample size, multiple assessment measures, and the possibility that patients who participated may have been more open to use of mobile health technology compared to patients who dropped out or chose not to participate. Future work should examine these relationships in larger samples of patients and patients with varying comfort levels with mobile technology. Also important to note is that medical-related factors such as illness severity/progression, medication effects, and cognitive limitations may limit a patient's ability to participate meaningfully in cognitive-behavioral coping skills training protocol; future research should consider this issue and work to identify those patients who are appropriate candidates and most likely to benefit from this treatment approach. Finally, given the significant difference in time to intervention completion but the absence of difference in outcomes (e.g., pain) between groups, it will be important to consider optimal time for intervention in future trials.

## 5. Conclusions

This small, randomized controlled pilot trial is one of the first trials to examine the accessibility and efficacy of mPCST intervention for cancer patients with pain compared to an in-person PCST intervention. Our findings suggest that pain coping skills training intervention that capitalizes on the advantages provided by mobile technologies is accessible to cancer patients with pain and is likely to lead to improved pain, self-efficacy for pain management, and other important pain-related outcomes. More work is necessary in this area to provide definitive information on the value of mobile health behavioral pain interventions.

## Figures and Tables

**Table 1:** Baseline demographic and medical data (*N* = 30).

	M	SD	%	*n*
Age	60	11		
Gender-female			50%	15
Marital status				
Single, widowed, divorced			21%	8
Married/life partner			79%	22
Education				
Less than high school			3%	1
High school diploma			14%	4
Some college			21%	6
Bachelor's degree or higher			62%	18
Race				
White			97%	29
Black			3%	1
Cancer type				
Breast			23%	7
Lung			23%	7
Prostate			46%	13
Colorectal			11%	3
Time since initial diagnosis (months)	41	55		
Stage at study entry				
0			3%	1
1			13%	4
2			17%	5
3			27%	8
4			40%	12
Comorbid disorders^a^				
Hypertension			39%	11
Heart disease			14%	4
Rheumatoid arthritis			4%	1
Osteoarthritis			21%	6
Diabetes			21%	6
Sciatica			8%	2
Emphysema, asthma, COPD			31%	8
Crohn's disease or irritable bowel syndrome			4%	1
Cancer treatments in last week^b^				
Chemotherapy			40%	11
Radiation			11%	3
Surgery			0%	0
Hormone therapy			30%	8
Vaccine			4%	1
Patient reported cancer therapy			28%	8

*Note*. ^a^10 participants reported more than one comorbid disorder; ^b^7 participants reported two or more treatments in the last week.

**Table 2:** Correlations between demographic and medical variables and study outcome variables at baseline.

	Age	Gender	Marital	Education	Race	Time diagnosed	Cancer stage	Comorbid disorders
Pain intensity	−.43^*∗*^	.20	.01	−.24	−.01	−.16	.21	.30
Pain catastrophizing	−.29	.12	.27	.04	.21	−.02	.33	.36
Pain self-efficacy	.21	.06	.03	.07	.04	.07	−.05	−.02
Physical functioning	−.28	.02	.15	.17	−.13	−.16	.14	−.01
Physical symptoms	−.01	.14	−.14	−.08	.10	−.20	−.15	.26
Distress	−.23	.49^*∗∗*^	−.11	−.21	.21	−.12	−.13	.05
Despair	−.13	.12	.05	−.11	.44^*∗*^	.12	.38^*∗*^	.40^*∗*^

*Note*. ^*∗*^ *p* < 0.05 and ^*∗∗*^ *p* < 0.01; gender code: 0 = male and 1 = female; marital code: 0 = not partnered and 1 = partnered; and race code: 0 = White and 1 = African American.

**Table 3:** Comparative pre- and postintervention data (*N* = 23).

	Before intervention		After intervention	
	M	SD	M	SD
Pain intensity				
Full group	4.19	1.86	2.68	1.98
mHealth	4.53	2.12	3.32	1.89
In person	3.86	1.61	2.05	1.95
Pain catastrophizing				
Full group	1.89	1.13	1.01	0.96
mHealth	1.64	1.33	1.42	0.99
In person	2.13	0.91	0.60	0.77
Pain self-efficacy				
Full group	48.76	18.40	60.32	27.81
mHealth	46.00	20.51	51.45	25.78
In person	51.30	16.72	68.44	28.16
Physical functioning				
Full group	5.08	2.37	3.94	2.51
mHealth	4.10	2.78	4.75	2.37
In person	5.98	1.52	3.20	2.51
Physical symptoms				
Full group	3.85	1.72	2.78	2.13
mHealth	3.27	1.51	2.80	1.0
In person	4.34	1.64	2.76	2.93
Distress				
Full group	2.13	1.72	1.75	2.18
mHealth	1.70	1.26	1.23	1.21
In person	2.52	2.04	2.27	2.81
Despair				
Full group	1.24	1.48	0.95	1.71
mHealth	1.05	1.31	0.70	0.80
In person	1.43	1.66	1.21	2.32

*Note*. All comparisons were nonsignificant.
